# Evaluation of Blood Biomarkers and Parameters for the Prediction of Stroke Survivors’ Functional Outcome upon Discharge Utilizing Explainable Machine Learning

**DOI:** 10.3390/diagnostics13030532

**Published:** 2023-02-01

**Authors:** Aimilios Gkantzios, Christos Kokkotis, Dimitrios Tsiptsios, Serafeim Moustakidis, Elena Gkartzonika, Theodoros Avramidis, Nikolaos Aggelousis, Konstantinos Vadikolias

**Affiliations:** 1 Department of Neurology, School of Medicine, University Hospital of Alexandroupolis, Democritus University of Thrace, 68100 Alexandroupolis, Greece; 2 Department of Neurology, Korgialeneio—Benakeio “Hellenic Red Cross” General Hospital of Athens, 11526 Athens, Greece; 3 Department of Physical Education and Sport Science, Democritus University of Thrace, 69100 Komotini, Greece; 4 AIDEAS OÜ, Narva mnt 5, 10117 Tallinn, Estonia; 5 School of Philosophy, University of Ioannina, 45110 Ioannina, Greece

**Keywords:** stroke, prognosis, functional outcome, poststroke disability, blood biomarkers, clinical data, artificial intelligence, explainability

## Abstract

Despite therapeutic advancements, stroke remains a leading cause of death and long-term disability. The quality of current stroke prognostic models varies considerably, whereas prediction models of post-stroke disability and mortality are restricted by the sample size, the range of clinical and risk factors and the clinical applicability in general. Accurate prognostication can ease post-stroke discharge planning and help healthcare practitioners individualize aggressive treatment or palliative care, based on projected life expectancy and clinical course. In this study, we aimed to develop an explainable machine learning methodology to predict functional outcomes of stroke patients at discharge, using the Modified Rankin Scale (mRS) as a binary classification problem. We identified 35 parameters from the admission, the first 72 h, as well as the medical history of stroke patients, and used them to train the model. We divided the patients into two classes in two approaches: “Independent” vs. “Non-Independent” and “Non-Disability” vs. “Disability”. Using various classifiers, we found that the best models in both approaches had an upward trend, with respect to the selected biomarkers, and achieved a maximum accuracy of 88.57% and 89.29%, respectively. The common features in both approaches included: age, hemispheric stroke localization, stroke localization based on blood supply, development of respiratory infection, National Institutes of Health Stroke Scale (NIHSS) upon admission and systolic blood pressure levels upon admission. Intubation and C-reactive protein (CRP) levels upon admission are additional features for the first approach and Erythrocyte Sedimentation Rate (ESR) levels upon admission for the second. Our results suggest that the said factors may be important predictors of functional outcomes in stroke patients.

## 1. Introduction

Despite significant breakthroughs in early detection, due to high-quality imaging and therapeutic interventions such as thrombolysis and thrombectomy, stroke continues to be a major cause of acquired disability in adults and the second leading cause of death worldwide, for individuals of all ages [1,2]. Approximately twenty-five percent of people over the age of twenty-five will experience a stroke in their lives and more than fifty percent of stroke survivors continue to have adverse outcomes, including chronic functional reliance and even death, despite the standard care provided in specialized stroke units. Therefore, it is evident that the development of new rehabilitation-improving therapies is necessary [3,4]. Currently, there are several data points about the factors that affect the prognosis and functional results of stroke. These variables pertain to the patients’ concurrent conditions, genetic and demographic traits, as well as the data collected during the acute phase of the stroke and connected to the stroke as an entity. More specifically, it is well documented and described in numerous studies that the vascular profile of such patients is determined by the presence of diseases such as hypertension, diabetes, hypercholesterolemia, a previous transient ischemic attack, atrial fibrillation, a history of ischemic heart disease (angina or myocardial infarction), internal carotid artery hemodynamically significant stenosis and peripheral arterial disease [5,6,7]. In terms of demography, age and gender have been identified as key prognostic risk factors. In addition, elevated CRP and creatinine values, the number of cardiovascular disease sites (cerebrovascular, coronary and peripheral arterial disease) and the presence of an infection at the onset of stroke appear to be independent risk factors for a high risk of early stroke recurrence during hospitalization [8,9,10,11]. Using MRI and, more specifically, the DWI sequence, which can be used to measure the cerebral infarct volume, it is further possible to anticipate the patient’s disability, thus serving as an independent predictor of the 90-day modified Rankin scale (mRS) outcome for neurologic disability in the acute ischemic stroke (AIS) [12].

To date, there have been no significant breakthroughs comparable to those noticed in acute stroke treatment. We understand what causes a stroke considerably better than what promotes recovery of function. Hence, it becomes vital to comprehend the underlying pathophysiological processes, in order to support rehabilitation. Rehabilitation therapies and services are essential for restoring patients’ abilities, independence and quality of life. However, a lack of consensus among measurements hinders the ability to fully maximize clinical findings and define a sufficient level of evidence for treatments, given the expanding availability of rehabilitation therapies. Thus, in order to study in depth the factors that influence patients’ recovery and to support the clinical choice regarding tailored treatment, a precise and comprehensive assessment is required [13,14,15].

In this direction, blood biomarkers have emerged as a useful diagnostic tool and a new method for accurately predicting functional outcomes following stroke [16], while several risk-prediction models have been developed thus far for detecting functional outcomes following an acute stroke. The majority of the latter base their prognosis on comparable input factors such as age, the severity of the first stroke, and comorbidities. However, none of these predictive models has been generally utilized in clinical practice, most probably due to implementation challenges [17,18,19,20]. As a result, the most difficult aspect of stroke rehabilitation research is the optimizing of rehabilitation regimens, based on an early prognosis [21,22]. A precise prognosis may aid in post-stroke discharge planning and the development of a more individualized rehabilitation program. However, it may be difficult to accurately predict the patients’ results, most particularly in the early post-admission period [23,24,25].

The far more recent rise in the respective studies’ interest might be ascribed to the growing complexity and quantity of data, as well as to the existence of multivariate data with heterogeneous origins, for which conventional approaches have proven incapable of delivering accurate results [22,26,27]. In recent years, machine learning (ML) methodologies have been widely used to address complex problems in a range of scientific fields, including medicine [28,29,30,31,32]. Machine learning is a subset of artificial intelligence in which a computer acquires knowledge from data, without explicit programming of rules. Machine learning algorithms identify patterns within data and use them to make predictions on new data. These models are beneficial for utilizing enormous datasets, due to the computers’ capacity to rapidly process vast volumes of electronic data. Where people are expected to become overwhelmed by ever-growing volumes and complexities of data inputs, machine learning models frequently thrive. To develop machine-learning solutions for clinical usage, one must have a solid grasp of clinical care, data science and implementation science. The idea that machine learning might quickly and drastically improve health care by automating routine processes and increasing clinical decision-making is enticing today. Finding effective machine-learned solutions necessitates careful consideration of poor classification and collection of health care data, as well as the complexity of clinical choices and workflows. In fact, machine learning has the potential to significantly improve health care. To genuinely assist the patients we serve, however, a disciplined, inclusive, collaborative and iterative approach to the development and use of new technologies is essential. Nonetheless, the practice of medicine will remain relatively unchanged at its foundation. Medicine entails communicating with patients and assisting them at, frequently, the most difficult phases of their life. These higher cognitive activities, such as understanding a patient’s goals and assessing competing priorities, are unlikely to be delegated to a computer. The future will likely be characterized by augmented intelligence, in which computers may become indispensable instruments for patient care, allowing physicians to dedicate more time to the patient care [33,34].

Hence, in its attempt to prevent strokes, the scientific community focuses on the creation of models that can anticipate their occurrence. In this context and since its implementation, artificial intelligence (AI) has played a key role in disease prevention and is now ubiquitous [35,36,37]; a growing number of studies indicate that the ML technique is more accurate in predicting stroke outcomes than statistical methods or scoring systems [38,39,40]. According to a recent review, numerous studies have been undertaken to generate models for stroke diagnosis [41,42,43] or predict treatment responses and clinical outcomes, with the aim of developing tailored rehabilitation regimens [44,45,46,47]. The emergence of machine learning (ML) and deep learning has facilitated the incorporation of a wide variety of structured data for the data-driven prediction of clinically meaningful outcomes in stroke patients [20,48,49].

To this end, the aim of the study at hand is to collect and analyze data from the hyperacute and acute phases of stroke, with the goal of developing an explainable machine learning model for predicting functionality at discharge, as measured by the mRS. Even though there is no commonly accepted stroke outcome measurement scale, we decided to use the mRS to assess the poststroke functional outcome of the patients, having considered the advantages of this scale. More specifically, the mRS is extensively used to assess residual disability and functional outcome in stroke patients. Both its reliability and its effectiveness in evaluating residual handicap in stroke patients have been thoroughly demonstrated. The mRS provides a rapid and straightforward evaluation of a patient’s stroke results, activities and social contribution, encompassing aspects of cognitive function, language, visual function and pain, that are not explicitly evaluated. Particularly, the 7-level mRS has several important strengths: its categories are simple and easily comprehended by both doctors and patients, its concurrent validity is established by a strong connection with measures of stroke pathology and agreement with other stroke scales, while its usage has delineated effective and ineffective acute stroke treatment. Having fewer levels than other stroke scales, the mRS may be less sensitive to change; yet, a single-point change on the mRS is clinically significant [50,51,52,53,54].

Explainable artificial intelligence (AI) is a type of AI, able to provide interpretable and transparent reasoning for its predictions or decisions. In this case, the aim is not only to accurately predict functionality at discharge but also to identify the variables from patients’ medical histories and admission biomarkers and parameters that contribute to the mRS result at discharge. This information can be used to support rehabilitation decision-making and improve patient outcomes by tailoring rehabilitation plans to the specific needs and characteristics of each individual patient. The use of explainable AI in this context is particularly important, as it can help healthcare professionals understand and trust the predictions and decisions made by the model, ultimately leading to better patient care.

## 2. Materials and Methods

### 2.1. Participants

This study included a total of 470 patients hospitalized with acute stroke at Korgialeneio—Benakeio “Hellenic Red Cross” General Hospital of Athens from July 2017 to June 2018. The study was approved by the Scientific Council of the hospital (protocol nr. 6673/08-03-2018). All patients were observed retrospectively until discharge. Stroke severity at admission was assessed using the National Institutes of Health Stroke Scale (NIHSS) and functional outcomes at discharge were assessed using the modified Rankin Scale (mRS). We determined a study population consisted of patients aged over 18, with ischemic or hemorrhagic stroke, without previous functional deficits (mRS before stroke, 0).

### 2.2. Data Description

In the present study, we collected patient data on a total of 35 different parameters or variables, as follows: demographic characteristics (age and gender), the type of stroke (ischemic or hemorrhagic), NIHSS scores and admission levels of systolic blood pressure, glucose, CRP and ESR. We also recorded data from patients’ medical history, including hypertension, smoking, diabetes, dyslipidemia, atrial fibrillation, previous stroke, previous myocardial infarction or coronary heart disease, history of heart failure, history of mechanical or bioprosthetic heart valve, history of alcoholism, history of taking antiplatelet drugs and history of taking anticoagulant drugs. We also recorded data from the first 72 h after a stroke, including intubation, initial diagnosis of hypertension, diabetes, dyslipidemia and atrial fibrillation, as well as total cholesterol, LDL, HDL, low T3 thyroid hormone, B12 vitamin, folic acid levels and the presence or absence of respiratory infection. For the same period of time, we also recorded stroke localization based on blood supply and hemispheric stroke localization according to CT or MRI brain imaging. At discharge, we assessed functional outcomes for each patient using the mRS.

### 2.3. Problem Definition

In this study, we consider the task of mRS grade prediction as a binary classification problem. Under this prism, we worked with two different approaches. In the first approach we divided the post-stroke patients into two categories: (i) Independent: Post-stroke patients with 0, 1 and 2 scores in mRS at discharge from hospital and (ii) Non-Independent: Post-stroke patients who scored 3, 4, 5 and 6 in mRS at discharge from hospital. In a second approach, we divided the employed post-stroke patients into two classes: (i) Non-disability: Post-stroke patients with 0 and 1 scores in mRS at discharge from hospital, who have no disabilities and (ii) Disability: Post-stroke patients who scored 2, 3, 4, 5 and 6 in mRS at discharge from hospital, who have mild to severe disabilities.

**1st Approach:** Independent for daily activities post-stroke patients versus non-independent for daily activities post-stroke patients, employing data from the baseline.

- Class A: involves 219 patients with 0, 1 and 2 mRS grades at discharge and
- Class B: comprises 247 patients with 3, 4, 5 and 6 mRS grades at discharge.

**2nd Approach:** Post-stroke patients without disabilities versus post-stroke patients with mild to severe disabilities, using data from the baseline.

- Class A: involves 153 patients with 0 and 1 mRS grades at discharge and
- Class B: comprises 313 patients with 2, 3, 4, 5 and 6 mRS grades at discharge.

For both approaches, the classification outputs 0 and 1 correspond to assignments to classes A and B, respectively.

### 2.4. Machine Learning Workflow Methodology

**Figure 1:**
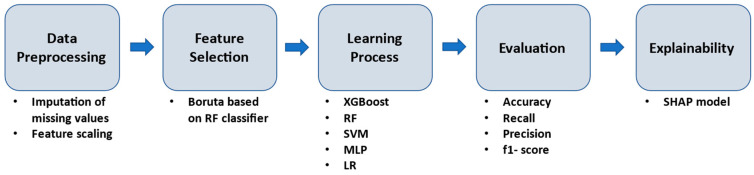
Workflow of the proposed methodology.

To identify robust biomarkers and parameters associated with the prediction of the functional outcome as assessed by mRS at discharge in the aforementioned approaches, a hierarchal ML pipeline was implemented (Figure 1). In the first stage, the data were normalized. The standardization of the features was performed by removing the mean and scaling to unit variance, in order to create a common basis for the feature selection stage and the learning process. For feature selection, we used the Boruta library, which is a wrapper technique based on a Random Forest classifier. This process ranked our clinical data by feature importance [55,56]. Five well-established classifiers were employed, including Random Forest (RF), XGBoost, Multi-Layer Perceptron (MLP), Support Vector Machines (SVMs) and Logistic Regression (LR). The purpose of employing five well-established classifiers was to determine their applicability to the specific problem. To minimize overfitting and maximize the performance of the ML models, hyperparameter tuning was applied. RF and XGboost classifiers belong to ensemble learning algorithms and were utilized as a result of their increased performance and speed [57,58]. MLP is a type of simple neural network that was included in this study because it has the ability to handle complex data and has a structure similar to the human brain [59]. In addition to MLP, we also employed SVMs and LR models in our proposed methodology [60,61]. Both of these models have been widely used in various applications and have shown good performance in classification tasks, which is why they were included in this study. SVMs are particularly useful when dealing with high-dimensional and complex datasets, while LR is a simple and efficient model that is particularly useful for binary classification problems.

We employed a 70% training and 30% testing data split to validate the proposed ML models. During the training phase, we used an internal 10-fold cross-validation to identify the consistent hyperparameters for each model. To balance the class distribution, we applied a random under-sampling reduction technique, in which the majority class was reduced to the same size as the minority class. To evaluate the performance of the proposed classifiers, we used several metrics including accuracy, f1-score, sensitivity (also known as recall) and precision. For the best-performing models, we also presented the confusion matrix. Finally, in order to understand the decision-making mechanism and the contribution of each selected feature in our medical task, we used Shapley Additive Explanations (SHAP) to interpret the output of the ML model. SHAP is a mini-explainer that provides a robust way to interpret ML models, which are often treated as black boxes [62,63,64]. By using SHAP, we become able to gain insights into the relative importance of each feature and the way it contributes to the overall prediction made by the model. This can be particularly useful for understanding the underlying mechanisms of the model and for identifying potential areas for improvement.

## 3. Results

In this section, we present the prediction performance results, the selected features and the explainability results for the best ML models, separately in each approach. We will provide detailed information on the performance of the models, the features that were most important in making predictions and the ways in which these features contributed to the overall prediction made by the model.

### 3.1. Prediction Performance

This sub-section focused on the testing performance results of the employed classifiers. These results are based on the classifiers trained on the first (1, 2, 3, etc.) most informative biomarkers. We will show the testing performance in relation to the number of selected biomarkers and compare it to the performance of the best model for each approach. The results will be presented separately for each approach.

**First Approach:** Independent and Non-Independent Categorization

**Figure 2:**
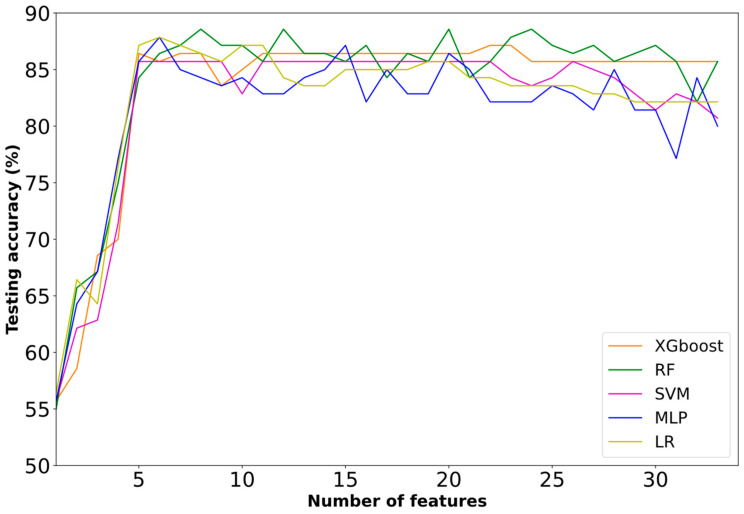
Learning curves based on the testing performance of the first approach.

In the first approach, all the competitive classifiers presented a rising trend regarding selected biomarkers in the first 2–6 features, with a maximum of 88.57% (RF classifier) at eight features (Figure 2). Furthermore, the inclusion of the additional biomarkers kept the performance of the XGBoost classifier constant. MLP and LR presented a decrease in accuracy with the addition of extra features.

Table 1 summarizes the best performance results of the employed ML models. The highest accuracy (88.57%) was achieved from RF at eight features. Furthermore, RF achieved 94.74% recall, 85.71% precision and a 90.00% f1-score. On the other hand, the lowest accuracy (85.71%) was achieved by the SVM classifier at five features.

**Figure 3:**
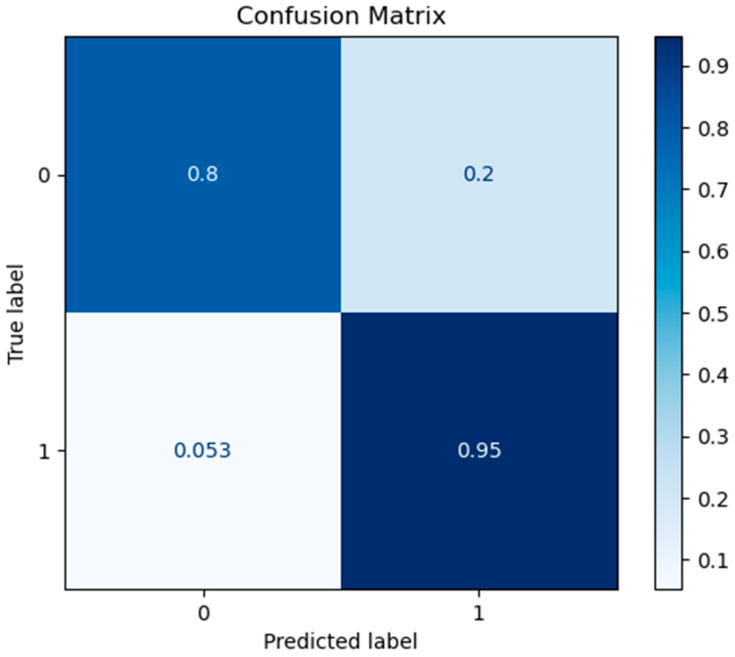
Confusion matrix for RF model at eight features.

In addition, the confusion matrix for the best ML model (RF classifier) is presented below in Figure 3.

**Second Approach:** Disability and Non-Disability Categorization

**Figure 4:**
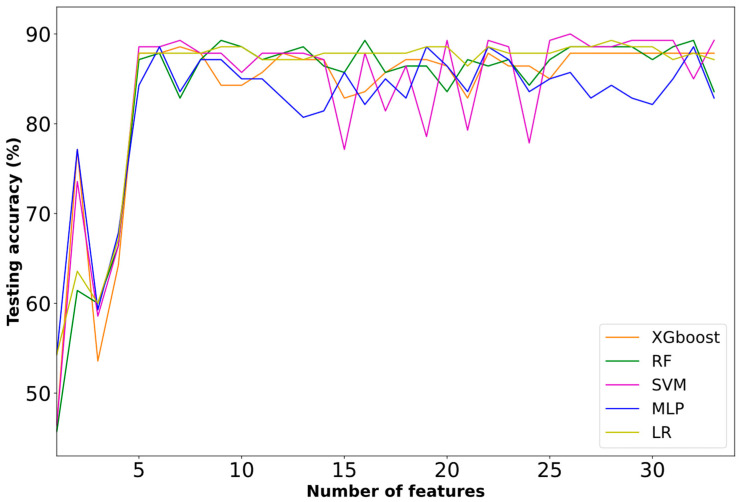
Learning curves based on the testing performance of second approach.

A corresponding trend to the aforedescribed first approach is observed in the prediction task for the binary problem of disability and non-disability. All the competitive classifiers presented a rising trend regarding selected biomarkers in the first 3–7 features, with a maximum of 89.29% at seven features (Figure 4). Furthermore, the inclusion of the additional biomarkers kept the performance in the majority of the classifiers constant. As observed, only the SVM presented non-constant performance with the additional biomarkers.

Table 2 demonstrates the best performance results of the employed ML models. The highest accuracy (89.29%) was achieved by SVM at seven features. Furthermore, SVM also achieved 89.47% recall, 94.44% precision and a 91.89% f1-score. On the other hand, the lowest accuracy (88.6%) was achieved by the XGBoost, MLP and LR classifiers at seven, six and nine features, respectively.

**Figure 5:**
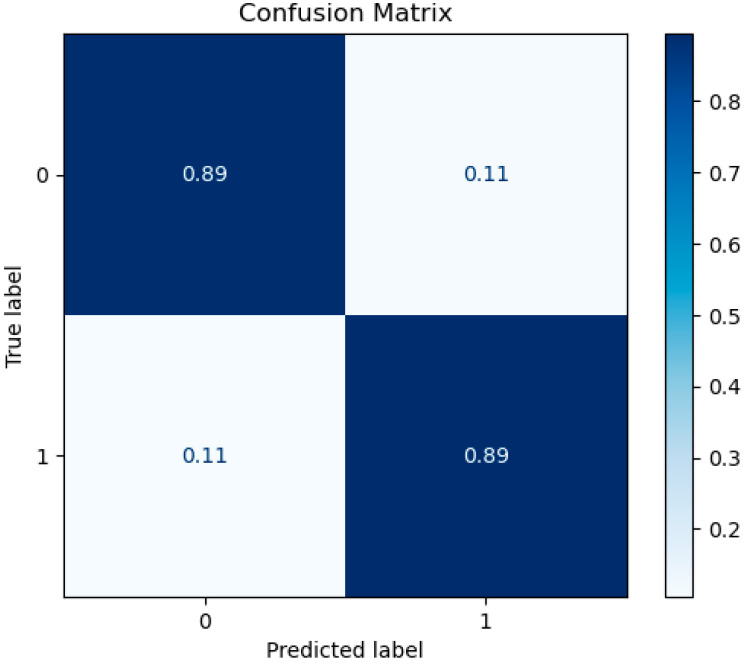
Confusion matrix for SVM model at seven features.

The confusion matrix for the best-performing machine learning model (SVM classifier) is shown below in Figure 5.

### 3.2. Selected Features

In this section, the importance of the selected features for both of the binary problems will be presented separately. As described in Section 2.4, we utilized the Boruta feature selection method to identify all relevant features in our dataset. Boruta is an all-relevant feature selection algorithm that compares the importance of a feature to the importance of a random shadow feature, using the RF algorithm to measure feature importance. The features were ranked based on their importance, and those with greater importance than the random shadow feature were considered relevant. The algorithm iteratively continues this process, until all the features have been either selected or rejected. As a wrapper method, Boruta uses the base estimator to compute feature importance and make a decision about feature selection. This technique is powerful for datasets with a large number of features and works well with both continuous and categorical features. In Table 3 and Table 4, the feature name, the description and the type of variable (numerical or categorical) are presented.

The feature importance results for both binary classification problems are presented in Table 3 and Table 4. These tables include the name of each selected feature and the type of the variable (whether it is numerical or not). These results provide insight into the factors that are most influential in determining the functional outcome of stroke patients at discharge, as assessed by the modified Rankin Scale. Understanding these factors can help inform the development of targeted rehabilitation strategies and interventions.

### 3.3. Explainability Analysis

**Figure 6:**
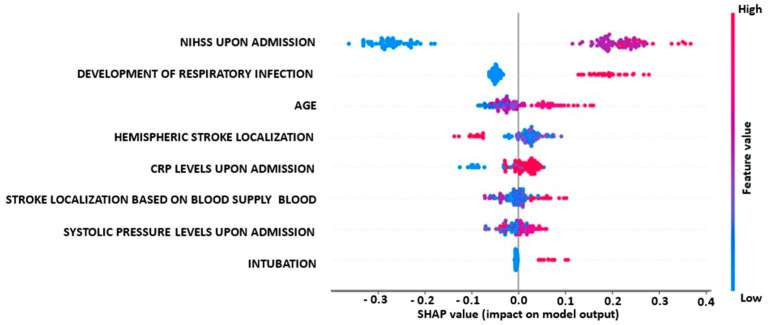
Biomarkers’ impact on RF model output for the prediction of mRS at independent vs. non-independent task.

In this section, the impact of different biomarkers and parameters on the output of the best-performing model (RF classifier) is evaluated. Figure 6 shows the relationships of these biomarkers and parameters with the binary outcome of being independent or non-independent in daily activities at discharge. The SHAP summary values of these biomarkers and parameters are displayed in descending order, with the most impactful features at the top. The color coding in Figure 6 indicates the extent of the impact on the model’s output, with red indicating the high values and blue indicating the low ones for each given observation.

According to the figure, NIHSS upon admission, the development of respiratory infection, age, CRP levels upon admission, stroke localization based on blood supply, systolic blood pressure levels upon admission and intubation all have a strong positive impact on the functional outcome for post-stroke patients, indicating that an increase in these values is closely associated with non-independence at discharge. On the other hand, hemispheric stroke localization has a negative correlation with non-independence at discharge, with left and bilateral localizations having better functional outcomes than most right hemispheric localizations.

**Figure 7:**
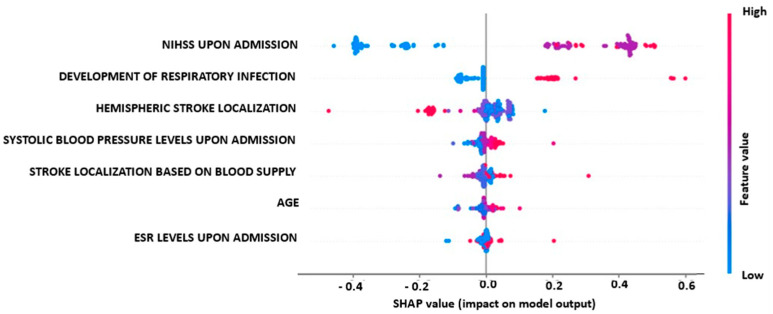
Biomarkers’ impact on MLP model output for the prediction of mRS at disability vs. non-disability task.

Figure 7 depicts the impact of biomarkers and parameters on the output of the best-performing model (SVM classifier) across test instances for the prediction task of disability versus non-disability. Factors such as NIHSS upon admission, the development of respiratory infection, systolic blood pressure levels upon admission, stroke localization based on blood supply and age have a high and positive impact on the pure functional outcome for post-stroke patients (e.g., the development of respiratory infection pushes the model output towards the disability class), thus indicating that the presence of respiratory infection is closely associated with mild to severe disabilities in post-stroke patients. On the other hand, hemispheric stroke localization is negatively correlated with the disability class of post-stroke patients, with left and bilateral hemispheric stroke localizations generally resulting in better functional outcomes at discharge compared to the majority of right hemispheric stroke localizations.

## 4. Discussion

In this study, having identified and utilized the aforementioned 35 parameters upon admission, in the first 72 h and from the medical history of patients with stroke, we attempted to develop an explainable machine learning model for the prediction of functional outcome at discharge. Under this prism, we worked with two different approaches, which resulted in age, hemispheric stroke localization, stroke localization based on blood supply, development of respiratory infection, NIHSS upon admission, CRP levels upon admission, systolic blood pressure levels upon admission, intubation and ESR levels upon admission being the more crucial and influential features that appear in determining the functional outcome of stroke patients at discharge. What follows is a literature-based discussion of the association between those relevant criteria, the functional outcome prognosis of stroke patients and the findings of our analysis, taking into consideration that, hitherto, researchers have separately evaluated each of the aforementioned factors but have not juxtaposed them with each other. In particular: age is considered the primary non-modifiable risk factor for stroke [65,66]. The mortality and morbidity rates as well as the functional recovery rates for elderly stroke patients are higher than those for younger patients [67]. In several previous epidemiological studies, age, along with baseline stroke severity as depicted in NIHSS, is another significant predictor of post-stroke outcome [68]. In the respective literature it is also claimed that as age increases, evidence-based stroke care guidelines are less likely to be followed [66,69,70].

Stroke localization, in which hemispheric detection or blood supply are involved, appears to have a complex relationship with the spatial distribution of the damage and its relevance to functional recovery. Several studies [71,72,73,74] have demonstrated that hemisphere lateralization has variable effects on functional outcomes. It is generally recognized that ischemic strokes affecting the dominant hemisphere, which in the majority of the population is the left- as opposed to the non-dominant or right hemisphere, can result in unique clinical syndromes and deficits depending on the regions affected. Multiple studies [74,75,76,77,78,79,80,81,82] have also shown that the right hemisphere’s involvement is predictive of inferior functional outcomes in patients with AIS. Given that the connection between lesion location, clinical impairments, recovery and functional outcome, as measured by mRS, is expected to be altered by the site of the lesion, differentiation is crucial [82,83,84,85,86,87]. On the other hand, there have also been reports indicating that hemisphere lateralization has no effect on functional outcomes and that there is no significant difference between left- and right-hemispheric strokes in terms of favorable result rates [72,73,83,84]. From the above, it is obvious that infarct location can have a significant impact on the prognosis of individuals, who have suffered an acute ischemic stroke; therefore, prediction models must account for it [85].

As highlighted in several studies, following a stroke, respiratory infection and, more specifically, pneumonia are closely related to an increased risk of poor outcome or mortality. Within the first three months following a stroke, two of every three cases of pneumonia occur in the first week, with the highest frequency occurring on the third day. Evaluating preventative measures for pneumonia is most effective during the first four days after a stroke. Pneumonia that develops later has also been associated with poor functional outcomes or mortality [86,87,88,89,90,91].

The NIHSS is a quantitative tool used to evaluate the severity of a stroke. It has been found to be a robust predictor of functional outcome 90 days after stroke. Low (<5) and high (>22) NIHSS cut-off points are highly predictive of positive (mRS 0-1) and negative (mRS 4–5 or death) outcomes, respectively. Results are less clear for intermediate initial NIHSS and symptom duration criteria. However, it favors anterior circulation stroke over posterior circulation stroke, resulting in larger results for the former. The mean NIHSS in the group with a favorable functional outcome is considerably lower for posterior circulation stroke than for anterior circulation stroke, highlighting the need to base acute stroke emergency management decisions on a comprehensive neurological examination, as opposed to a single scale, especially in the cases where posterior circulation stroke is in question [92,93,94,95].

Studies have shown that elevated CRP levels in the early stages of ischemic or hemorrhagic strokes are related to stroke severity, poor clinical outcomes and long-term prognosis. In particular, after an ischemic stroke, CRP has been found to be an independent predictor of long-term death [96,97].

Hypertension is a well-documented modifiable stroke risk factor. J- and U-shaped curves have been seen between systolic blood pressure (SBP) and clinical outcomes in acute ischemic stroke patients (a satisfactory clinical result at 3 months was construed as an mRS score between 0 and 2); both low and high SBP have been associated with adverse outcomes (mRS 3–6). According to a number of studies, elevated blood pressure after a stroke may increase infarct volume and is associated with a poorer clinical outcome (mRS 3–6). There are other reports indicating that a higher diastolic blood pressure (DBP) at admission is predictive of in-hospital mortality among patients with acute ischemic stroke. Elevation in mean arterial pressure (MAP) is indicative of increased mortality over the long term. Higher entry blood pressure (BP) in patients suffering from acute ischemic stroke, who were treated with endovascular therapy (EVT), is associated with reduced odds of effective reperfusion and poor clinical outcomes. Other studies affirm that, in patients with AIS, a low SBP on admission is associated with an increased risk of in-hospital mortality and comorbidities such as heart failure, gastrointestinal bleeding and sepsis [98,99,100,101,102,103,104,105,106].

Regarding intubation in the hyperacute phase of stroke or in the early days after stroke, recent research, despite being retrospective, suggests that intubation is associated with an increase in mortality. Moreover, various studies argue that poor outcomes may be associated with an increased risk of aspiration pneumonia and sepsis in intubated patients as opposed to non-intubated patients [107,108,109,110,111].

Lastly, the levels of ESR at admission should not be used as a direct predictors of inflammation in ischemic stroke. While other markers of inflammation, such as CRP, fibrinogen and triglycerides, may be elevated in ischemic stroke patients, ESR levels alone may not be sufficient to identify patients with persistent ischemic stroke episodes. It is worth noting that ischemic stroke patients often have a wide range of risk factors and it is possible that they may have a pre-existing proinflammatory procoagulant state, which could contribute to the shift in ESR values shortly after a cerebral infarction, with the exception of hematological parameters [112].

In our study, for the first approach, the biomarkers’ and parameters’ impact on the best-performing model’s (RF classifier) output across test instances for independent vs. non-independent prediction classes, with an accuracy of 88.57%, revealed that NIHSS upon admission, the development of respiratory infection, age, CRP levels upon admission, stroke localization based on blood supply, systolic blood pressure levels upon admission and intubation show a high and positive impact on the pure functional outcome for post-stroke patients. On the other hand, hemispheric stroke localization is negatively correlated with non-independent post-stroke patients. More specifically, hemispheric stroke localizations such as left and bilaterally have better functional outcomes at discharge than the majority of the right hemispheric stroke localizations. For the second approach, the biomarkers’ and parameters’ impact on the best-performing model’s (SVM classifier) output across test instances for the disability vs. non-disability prediction task, with an accuracy of 89.29%, revealed that NIHSS upon admission, the development of respiratory infection, systolic blood pressure levels upon admission, stroke localization based on blood supply and age show a high and positive impact on the pure functional outcome for post-stroke patients. On the other hand, hemispheric stroke localization is negatively correlated with the disability class post-stroke patients. In particular, hemispheric stroke localizations such as left and bilaterally have better functional outcomes at discharge than the majority of the right hemispheric stroke localizations.

### 4.1. Limitations

Our research was concluded under the assumption of the following limitations: Although the mRS is the most commonly used outcome scale in stroke research, it is primarily dictated by motor impairment and is rather insensitive to cognitive impairment. As a measure of global functional status, the mRS is highly dependent on ambulatory status and lacks sensitivity for deficits in other domains, such as language or neglect, which influence functional outcomes. Due to the inherent limitations of the mRS, disparate methods are required to evaluate functional results. In addition, due to the fact the study at hand is retrospective, namely the database was created before the widespread application of intravenous thrombolysis to patients with ischemic stroke and the mechanical thrombectomy era, our results require further replication in a prospective population. Despite our confident belief that the same parameters will remain able to predict the functional outcome, we expect that the proportion of patients with severe or moderate stroke is likely to be reduced, resulting in a superior functional recovery, given the administration of intravenous thrombolysis to a number of stroke patients. Finally, it shall be noted that the data pertain to one hospital, even though the Neurological Clinic handles a sufficient number of stroke patients of all ages—a feature that distinguishes it from the majority of neurological clinics in Greece. Hence, it should finally be taken into consideration that the sample represents particular geographic and socioeconomic features.

### 4.2. Future Work

In future work, we will employ state-of-the-art ratios of biomarkers and parameters for the long-term prediction (≥3 months) of the functional outcome at the acute phase of stroke patients utilizing robust feature selection techniques and explainable machine learning (XAI) techniques. An external validation dataset will be employed in order to assess the best model’s reproducibility and generalizability. In addition, we intend to create a database in which Brain Reserves, such as brain volume and leukoaraiosis, will be examined and evaluated.

## 5. Conclusions

In conclusion, this study identified 35 parameters upon admission, in the first 72 h and from the medical history of patients with stroke and attempted to develop an explainable machine learning model for the prediction of functional outcome at discharge using the mRS as a binary classification problem. Two approaches were employed and competitive classifiers showed a rising trend in relation to selected biomarkers in the first 2–6 features of the first approach and in the first 3–7 features of the second approach. The maximum accuracy was achieved at eight and seven features, respectively, with six of the features being common to both approaches. Up to today, the predictive models for post-stroke outcome that have been developed using machine learning have focused on large-vessel ischemic strokes that underwent mechanical thrombectomy, and the functional result of patients was assessed three months after the stroke [29,38,113,114,115]. To the best of our knowledge, this study is the first attempt to collect data during the acute phase of stroke in conjunction with the detection of parameters from the medical history of these stroke patients and to analyze the data using machine learning, in an effort to develop a prognostic model of the functional outcome of the patients upon hospital discharge, thereby potentially contributing to timely, but also more accurate and individualized, diagnosis and treatment. This information can also be used to support rehabilitation decision-making and improve patient outcomes by tailoring rehabilitation plans to the specific needs and characteristics of each individual patient. While the results of this study appear to be promising in predicting functional outcome in stroke patients, it is important to note the limitations of the mRS as an outcome measure and the retrospective nature of the study. Further replication in a prospective population with the inclusion of intravenous thrombolysis and mechanical thrombectomy is necessary to confirm the robustness of these findings. Withal, this study’s sample size was restricted to a single institution and may not be representative of stroke patients in different geographic and socioeconomic contexts.

## Figures and Tables

**Table 1:** Best scores of the competitive classifiers.

Classifiers	Accuracy (%)	Recall	Precision	f1-Score	Num of Features
XGboost	87.14	96.05	82.96	89.02	22
**RF**	**88.57**	**94.74**	**85.71**	**90.00**	**8**
SVM	85.71	93.42	82.56	87.65	5
MLP	87.86	96.05	83.91	89.57	6
LR	87.86	93.42	85.54	89.03	6

In bold the model with the highest accuracy.

**Table 2:** Best performance results of the employed ML models.

Classifiers	Accuracy	Recall	Precision	F1-Score	Num of Features
XGBoost	88.59	87.35	95.41	91.21	7
RF	89.27	89.45	94.43	91.88	9
**SVM**	**89.29**	**89.47**	**94.44**	**91.89**	**7**
MLP	88.57	87.37	95.42	91.22	6
LR	88.56	87.36	95.40	91.23	9

In bold the model with the highest accuracy.

**Table 3:** Ranking according to the feature significance of the selected characteristics from the best ML model (RF) at the binary problem independent vs. non-independent.

Ranking	Features	Type
1	Age	Categorical
2	Hemispheric stroke localization	Categorical
3	Stroke localization based on blood supply	Categorical
4	Development of respiratory infection	Categorical
5	NIHSS upon admission	Categorical
6	CRP levels upon admission	Categorical
7	Systolic blood pressure levels upon admission	Categorical
8	Intubation	Categorical

**Table 4:** Ranking according to the feature significance of the selected characteristics from the best ML model (RF) at the binary problem disability vs. non-disability.

Ranking	Features	Type
1	Age	Categorical
2	Hemispheric stroke localization	Categorical
3	Stroke localization based on blood supply	Categorical
4	Development of respiratory infection	Categorical
5	NIHSS upon admission	Categorical
6	Systolic blood pressure levels upon admission	Categorical
7	ESR levels upon admission	Categorical

## Data Availability

The dataset generated and/or analyzed during the current study is not publicly available.
